# Social niche construction and evolutionary transitions in individuality

**DOI:** 10.1007/s10539-015-9505-z

**Published:** 2015-11-19

**Authors:** P. A. Ryan, S. T. Powers, R. A. Watson

**Affiliations:** Institute for Life Sciences, Electronics and Computer Science, University of Southampton, Southampton, UK; Department of Ecology and Evolution, University of Lausanne, Lausanne, Switzerland

**Keywords:** Major transitions in evolution, Extended Evolutionary Synthesis, Dialectical biologist, Niche construction, Interactionism, Evolution of cooperation, Game theory, Explanation

## Abstract

Social evolution theory conventionally takes an externalist explanatory stance, treating observed cooperation as explanandum and the positive assortment of cooperative behaviour as explanans. We ask how the circumstances bringing about this positive assortment arose in the first place. Rather than merely push the explanatory problem back a step, we move from an externalist to an interactionist explanatory stance, in the spirit of Lewontin and the Niche Construction theorists. We develop a theory of ‘social niche construction’ in which we consider biological entities to be both the subject and object of their own social evolution. Some important cases of the evolution of cooperation have the side-effect of causing changes in the hierarchical level at which the evolutionary process acts. This is because the traits (e.g. life-history bottlenecks) that act to align the fitness interests of particles (e.g. cells) in a collective can also act to diminish the extent to which those particles are bearers of heritable fitness variance, while augmenting the extent to which collectives of such particles (e.g. multicellular organisms) are bearers of heritable fitness variance. In this way, we can explain upward transitions in the hierarchical level at which the Darwinian machine operates in terms of particle-level selection, even though the outcome of the process is a collective-level selection regime. Our theory avoids the logical and metaphysical paradoxes faced by other attempts to explain evolutionary transitions.

## Social evolution theory employs an externalist explanatory stance

In biology, the externalist explanatory stance takes it that the properties of biological entities are to be explained by factors largely or entirely external to those entities (Spencer 1864; Lack 1947; Simon 1981; Williams 1966; Brandon 1990; Godfrey-Smith 1996). Social evolution theory typically employs an externalist explanatory stance. This sort of explanation starts by noting the existence of some form of biological cooperation or altruism. This behaviour is taken to stand in need of explanation because, on first examination, it is inconsistent with the predictions of evolutionary theory—Darwin’s ‘one special difficulty’. Cooperation is paradoxical for evolutionary theory, until we notice the presence of some factor that explains why the observed behaviour is adaptive after all. In many well-studied cases, the factor used to explain the social behaviour is genetic relatedness between actor and recipient (Hamilton 1964). In others, it is explained by the presence of iterated interaction between re-identifiable individuals (Trivers 1971; Axelrod and Hamilton 1981). In still further cases, among symbiotic mutualists, it is explained by vertical transmission (co-dispersal) (Ewald 1987) or partner discrimination (Noë 2001; Kiers et al. 2011; Archetti et al. 2011). The explanatory pattern is linear and proceeds in only one direction:^1^ there is some factor external to the organism that explains some social trait (ST) of the organism as an evolutionary response to that factor. In the absence of the external factor, we would not expect cooperation to be evolutionarily stable. In the presence of the factor, we understand cooperation to be an adaptive response to it. Different classes of cooperation are explained by different classes of external factors. For example, within-colony kinship might be used to explain reproductive altruism in eusocial hymenoptera (Bourke and Franks 1995), while life-history considerations pertaining to vertical transmission are used to explain ongoing stability of mutual cooperation in the symbiosis between the aphid *Acyrthosiphon pisum* and its bacterial endosymbiont *Buchnera aphidicola* (Wernegreen and Moran 2001). Different cooperative or altruistic behaviours have different explanations and those explanations each invoke some external factor which is itself unexplained.

## Can we give a general Darwinian explanation for the origin of factors enabling cooperation?

The factors invoked to explain cooperation^2^ are rich and varied. Can we give a general Darwinian explanation for the *origin* of those factors? This line of inquiry could go one of a few ways. We might find that, in each case, the factors to be explained arose in a manner for which no Darwinian explanation is (or is yet) available. Alternatively, we might find that, while each case admits of Darwinian explanation, all the cases are very different. Perhaps there are no general principles to be discovered about the evolution of these things and the best we can hope for is a ‘patchwork’ of explanations (Dupré 1995; Cartwright 1999). After all, explanations involving relatedness, reciprocity and group selection all look rather different. Or perhaps, as we contend, a general explanation is available—one that offers conceptual unification across the apparently disparate cases. That is the motivation behind social niche construction theory (Powers 2010; Powers et al. 2011).

## Social niche construction theory employs an interactionist explanatory stance to reciprocally explain both cooperation and the factors enabling it

Social evolution theory tries to explain the evolution of social behaviours (Hamilton 1964; Wilson 1975a; Bourke 2011); niche construction is any process in which organisms modify their own environment in such a way as to influence the conditions of their own evolution (Odling-Smee et al. 2003; Laland and Sterelny 2006). The term ‘social niche construction’ is intended to denote the application of niche construction theory to social evolution.

In the dialectical spirit of Lewontin (1985) and the Niche Construction theorists (Odling-Smee et al. 2003), social niche construction theory employs an explicitly interactionist explanatory stance (Oyama et al. 2000, 2001; Barberousse et al. 2009). The central idea is that biological entities are both the subject and object of their own social evolution. The advantage of this sort of thinking is that it allows us to explain changes in terms of their effects without running into any metaphysical problems about the order of cause and effect, it avoids explanatory postponement and it dissolves the apparent paradox facing attempts to explain how natural selection acting at some level in the biological hierarchy could shift that very process to a higher level from below. We think our interactionist explanatory stance corresponds more closely to the causal structure of the evolutionary processes we seek to explain.

We introduce the following terminology:

- *Particle, collective* Lower and higher level entities in a two-level part-whole hierarchy.
- *Social trait (ST)* A trait that affects the fitness of individuals other than the actor, sometimes having values appropriately labelled ‘cooperate’ or ‘defect’ (e.g. Kropotkin 1902; Bourke and Franks 1995; Crespi 2001; Calcott 2008).
- *Social niche* The selective context in which social behaviour occurs, affecting the strength and direction of selection on it. In game theoretic terms, the social niche is the effective game being played, once all relevant factors have been taken into account. (Relevant factors include the underlying game, any social niche modifiers (SNMs) that may be present and the frequencies of various strategies in the population.)
- *Social niche modifier (SNM)* A trait that alters the effective game being played by its bearers, causing it to differ from the counterfactual game they would have been playing if the SNM had not acted. Examples include factors such as population structure, relatedness, punishment, policing and side-payments (see Table 1 for many more).
- *Social niche construction* A circular process in which organisms modify their own social niche in such a way as to influence the conditions of their own social evolution.

Social niche and SNM are inter-defined. The SNM trait is called a ‘modifier’ because it changes the social niche that *would have* obtained had it not been in operation. It is by the action of SNM traits that individuals can (partly) construct their own social niche and so the circumstances of their own social evolution. Policing, punishment, side-payments and relatedness among interaction partners are examples of SNMs that can arise as a result of individuals’ social niche modifying traits. It is important for their evolution that SNMs alter the effective game being played in their bearers’ interactions, not for the whole population.

Assortment can be an important SNM. Consider a situation where a population faces a public goods game instantiating a Prisoners’ Dilemma and is freely-mixed. Here the social niche is a Prisoners’ Dilemma, where defection is the evolutionarily stable strategy (ESS). Now consider a contrasting situation where a population faces the very same public goods game but in the presence of a population structure, such that individuals interact only with clones of themselves. The effective game being played in this modified social niche is a Harmony Game, where cooperation is ESS.

In another example, consider punishment as a SNM (Boyd et al. 2010). Suppose again that a population faces a public goods game instantiating a Prisoners’ Dilemma. In the absence of any SNM, the social niche is a Prisoners’ Dilemma and defection is the ESS. In the presence of punishment as a SNM, any temptation to defect is reduced by probable punishment, wiping out any gain that might be made from unilateral defection. In this social niche, cooperation is ESS even though defection would have been ESS if the SNM of punishment had not been in force.

It should now be clear what we mean when we say that the social niche is the *effective* game being played, once relevant factors have been taken into account. There is an important distinction between the social niche actually encountered and the social niche that would have been encountered in the absence of those relevant factors. We list a large number of putative examples of SNMs in Table 1 in the following section.

**Table 1:** Examples of structural feature of biological world that function as social niche modifiers for populations of entities interacting in their presence

Structural feature of biological world	Role as a social niche modifier
Suppression of segregation distorters in diploids (Maynard Smith 1958; Leigh 1971, 1991)	Yields fair meiosis, which avoids intragenomic conflict by placing the alleles at each locus on a diploid genome ‘in the same boat’ with regard to their chances of reproductive success right up until the moment segregation occurs (Haig and Grafen 1991). Gene and genome fitness interests are aligned in the presence of fair meiosis and not aligned without it
Obligate co-dispersal of mitochondria and chloroplast in eukatyotic cells	Vertical transmission means both partners meet a shared reproductive fate. This aligns the fitness interests of both parties in the symbiosis (Bergstrom et al. 2003)
Obligate co-dispersal of mycetocyte bacteria (operating in the gut) with their insect hosts. In many species, including cockroaches, transmission occurs in the ovaries (Douglas 1989)	Vertical transmission means both partners meet a shared reproductive fate. This aligns the fitness interests of both parties in the symbiosis (Ewald 1987)
Obligate co-dispersal of endophytic fungi with their symbiotic grasses and sedges (Clay 1990)	Vertical transmission means both partners meet a shared reproductive fate. This aligns the fitness interests of both parties in the symbiosis
Uniparental inheritance of mitochondrial DNA (Birky 1995, p. 149)	Avoids conflict that might occur if there were cytoplasmic chimerism in eukaryote cells (Burt and Trivers 2006, p. 149); also causes nuclear-cytoplasmic conflict over sex ratio (Schnable and Wise 1998)
Unicellular life-history bottlenecks (Dawkins 1982)	Alignment of cellular fitness interests in multicellular organisms, due to their clonal relatedness (Dawkins 1982)
Germline sequestration in metazoans (Buss 1987)	Denies heritability to selfish cell lineages arising in the soma. The inclusive fitness interests of somatic cells are then best served by supporting the reproduction of the germline cells, rather than attempting to reproduce directly (Michod 2006; Bourke 2011)
Apical meristem topology in vascular plants (Klekowski 1988)	Denies heritability to selfish cell lineages arising outside the apical initials. The inclusive fitness interests of somatic cells are then best served by supporting the reproduction of the apical cells, rather than attempting to reproduce directly
Allorecognition mechanisms in benthic tunicates (Grosberg 1988)	Avoids threat of parasitism (free-riding on club goods) that would be present if genetically unlike colonies merged freely
Allorecognition mechanisms in anenomes (Ayre and Grosberg 2005)	Avoids threat of parasitism (free-riding on club goods) that would be present if genetically unlike colonies merged freely
Self/nonself discrimination in filamentous fungi (Glass et al. 2000)	Avoids threat of parasitism (free-riding on club goods) that would be present if genetically unlike colonies merged freely
Cell-cycle synchronization in myxomycetes (Buss 1987, p. 130)	Turns potentially defector mutations (that increase cell fitness while decreasing plasmodium fitness) into ordinary deleterious mutations (that decrease both cell fitness and plasmodium fitness)
Kin-recognition mechanisms in cellular slime molds such as *Dictyostelium discoideum* (Mehdiabadi et al. 2006)	Avoids threat of free-riding that would be present if genetically unlike cells merged freely. High relatedness during the aggregation phase of the lifecycle enables cooperative division of labour between stalk and fruiting-body roles, both of which are necessary for successful reproduction (Bourke 2011)
Mechanisms of policing, punishment and coercion in eusocial insect societies (e.g. Ratnieks 1988; Wenseleers et al. 2004)	In the presence of these social niche modifiers, the inclusive fitness interests of workers are best served by supporting the reproduction of the colony (through the queen), rather than attempting to reproduce directly. Policing and punishment modify social niche without modifying assortment
Competition for scarce resources in demes of red grouse (Wynne-Edwards 1962)	No social niche modifier is mentioned in this example. Selfish behaviour is the default case, in no need of special explanation. Even though it might be possible to raise the carrying capacity if individuals exercised consumption restraint, this does not happen. This is because the fitness cost of such restraint is borne fully by the individual exercising it, while the benefit arising from it is enjoyed by the whole group [i.e. a Tragedy of the Commons (Hardin 1968)]. The effective game being played in the social niche is a Prisoners’ Dilemma. The unmitigated conflict of interests means cooperation (in the form of reproductive restraint) is not a fit strategy in such a social niche

The first five examples are implicated in egalitarian transitions. The other examples are implicated in fraternal transitions (Queller 1997)

Social niche construction is a circular process in which organisms modify their own social niche in such a way as to influence the conditions of their own social evolution. If a population varies in its social niche modifying trait then it is possible that not all individuals in the population experience the same social niche. If some focal set of individuals (minimally two) locally modify their social niche in a pro-social manner, then this can yield a change in the level of cooperation among those focal individuals (raising it above the level of cooperation among the wider population). The benefits of this increased cooperation increase the fitness of the bearers of the social niche modifying trait. (Note that this is not because there is direct selection on the SNM but because it is correlated with the ST—and the ST, in the locally modified social niche, confers a fitness advantage on the bearer.) There is a circularity here that warrants emphasis:

- a pro-social allele at the SNM locus enables higher levels of cooperation at the ST among its bearers and selection responds to this;
- a higher level of cooperation (ST) leads to higher fitness for the bearers of the pro-social allele at the (linked) SNM locus;
- repeat.

In this way, runaway selection on a linked pair consisting of an initially-rare mutant pro-social allele at the SNM locus and an initially-rare mutant cooperative allele at the ST locus can potentially invade a population bearing a wild-type SNM allele that does not enable cooperation.

Social niche construction theory predicts that whenever we find cooperative behaviour in the biological world, we expect to find co-evolved mechanisms supporting it. Without the mechanism the cooperation would not be evolutionarily stable and without the cooperation the mechanism would have no (adaptive) explanation. In “Social niche modifying traits (SNMs)” section we list many structural features of the biological world that we suggest might plausibly have evolved as SNMs in a process something like that sketched out here (e.g. the self/nonself discrimination mechanisms in filamentous fungi, the apical meristem topology of vascular plants and the obligate co-dispersal of endophytic fungi with their symbiotic sedges).

A number of authors have previously suggested, in general terms, that some sort of runaway social selection between population structure and social behaviour must be at work when we see the evolution of cooperation in nature (Breden and Wade 1991; Thompson 2005; Santos et al. 2006a; Rosas 2010; Van Dyken and Wade 2012; Clarke 2014; Sober and Wilson 1998, p. 97). Michod and Roze (1999) investigated the interplay of social behaviour with a modifier locus (that either imposed a bottleneck or policing) but they built collective-level selection into their model as one of its assumptions (we want to explain how collective-level selection gets started). There have also been a number of more game-theoretic studies investigating the effects of allowing the underlying game, usually a Prisoners’ Dilemma, to be changed by the players. Some involve individuals modifying the payoff matrix directly (Worden and Levin 2007), effectively modifying the payoff matrix by introducing side payments (Akçay and Roughgarden 2011), or modifying the payoff matrix by modifying assortment by enabling adaptive linking in a network setting (Pacheco et al. 2006a, b; Suzuki et al. 2008; Van Segbroeck et al. 2009; Cao et al. 2011; Kojima et al. 2012). See also Skyrms (2004). We attempt to construct a broad framework in which these studies can be seen as special cases.

Due to its emphasis on the role of constructive processes in evolution and on reciprocal causation, we view social niche construction theory as belonging to the emerging body of ideas known as the extended evolutionary synthesis (Laland et al. 2015). Social niche construction is a very general process we invoke to explain all stable forms of biological grouping (e.g. stable grouping of genes on chromosomes or cells in multi-cellular organisms). Social niche construction is therefore distinct from ‘cultural niche construction’, a process of interest in the study of human evolution, whereby human cultural traits can modify the strength and direction of selection on human genes (e.g. Laland et al. 2001; Borenstein et al. 2006). Recall that the ‘ST’ in social niche construction is defined as a behaviour that evolves because it affects the fitness of others in addition to the actor (e.g. altruism, mutual cooperation or selfishness) and is not to be confused with the societal or cultural traits of interest in the study of human evolution. In this paper we focus on cases of social niche construction where the ST and the social niche modifying trait are both genetic (and both vertically transmitted) and we do not discuss any human examples. However, we should point out that some cases of human cultural niche construction might also be cases of social niche construction (e.g. Powers and Lehmann 2013).

We should also note that SNMs making the conditions for cooperation less favourable could also exist but are of less interest to us because they do not have the same potential to drive upward changes in the level of selection and individuality. For a general mathematical treatment of social niche construction exploring the full range of theoretical possibilities, see Jackson and Watson (2015).

## Social niche modifying traits (SNMs)

When we examine a complex social group [i.e. collective life-form] we frequently see, like tourists watching a ceremonial changing of the guard, features that make sense only as the products of a more turbulent past. (Bourke 2011, p. 194)

Biological examples of social niche modifiying traits include old favourites such as life-history bottlenecks, early-segregating slowly-dividing germlines and worker-policing in social insects. However, rather than special cases or rare curiosities, we follow Bourke (2011) in interpreting a very wide range of structural features of the biological world as playing the functional role of SNMs, ubiquitous wherever complex adaptation is in evidence. In a multi-level setting, SNMs change the effective game being played between particles, moving particle and collective fitness interests into greater alignment. What is new here is not the idea *that* collective life-forms have mechanisms that ameliorate or avoid internal conflict (Michod and Herron 2006; Queller and Strassmann 2009) but to view the *evolution* of such mechanisms as admitting of a general theoretical treatment.

Collective action can be divided into two broad categories, the first involving the coming together of like kinds and the second involving the coming together of unlike kinds—in both cases to enjoy some mutual benefit that could not be accessed through solitary action (Mill 1848, Book I, Chap. VIII). The first case includes colonial organisms and can sometimes lead to ‘fraternal’ evolutionary transitions. The latter case includes inter-specific symbioses and can sometimes lead to ‘egalitarian’ transitions (Queller 1997). Social niche modifying traits also fall into two corresponding categories.

The first category involves like-kinds coalescing into higher-level units that, in extreme cases (fraternal transitions) come to be evolutionary units in their own right. These cases rely on *genetic relatedness* between the coalescing entities to align fitness interests. We understand the pathway to the fraternal transitions in terms of the evolution of SNMs affecting the relatedness between social partners (e.g. kin recognition or population viscosity). Relatedness is a very common realizer of the assortment of social behaviours. Mutualistic symbioses and, in extremis, egalitarian transitions involve unlike-kinds coming together. Egalitarian transitions rely on both parties to the coalition retaining their ability to reproduce, albeit within a mechanism that ensures they each do so only if the other does also. This forced *shared reproductive fate* aligns the fitness interests of both parties (Ewald 1987; Bourke 2011). We understand the pathway to the egalitarian transitions in terms of the evolution of SNMs affecting the obligate co-dispersal of both partners (sometimes called ‘vertical transmission’). Other SNMs, such as partner-discrimination and sanctioning may also be important for maintaining the positive assortment of cooperative behaviour in inter-specific mutualisms with horizontal transmission (Noë 2001; Sachs et al. 2004; Kiers et al. 2011; Archetti et al. 2011). In both the fraternal and egalitarian cases, while the unmodified social niche may feature conflict between particle and collective interests, the modified social niche finds those fitness interests brought into alignment.

## Varieties of social niche: When does it pay to be nice?

To understand the evolution of SNMs, it is necessary to understand the social niches they are modifying. Social niches that are ‘red in tooth and claw’, involving straightforward competition or predation are usually taken to be the default case and require no special explanation (pace Roughgarden 2009). In these cases, selfish behaviour is evolutionarily stable. An individual can gain only at the expense of another and there is no opportunity to increase overall social welfare. Things start to get interesting when we turn to the social niches that represent a cooperative dilemma (Dawes 1980). Here, there is an opportunity to avail of new fitness benefits available only through collective action (Olson 1965; Calcott 2008). However, such collective action is undermined by conflicts between individual and collective interests (Maynard Smith 1988). Individually rational behaviour leads to outcomes that are not the best collective outcome (Macy and Flache 2002). In cases such as this, we can usefully employ concepts from game theory to describe, categorise and explain the properties of a social niche. It is also in these cases that social niche construction has something new to offer because it can dissolve the apparent paradox that usually blocks attempts to explain, in terms of individual-level selection, how group-beneficial outcomes can arise in spite of the presence of a cooperative dilemma.

### Analysis of collective action between like-kinds using the T–S plane

In the remainder of this section, we present a minimally technical analysis of social niche construction among like-kinds. The like-kinds case has the advantage of greater simplicity of exposition and tractability of analysis, as we can model such collective action problems as the linear aggregate of payoffs in a pairwise two-player two-strategy symmetric game^3^ (Hamilton 1975). However, we emphasise that our general claims about social niche construction are intended to be understood more broadly than the detailed treatment we offer for cooperation among like-kinds and fraternal transitions in this part of the paper.

The games we use to model social niches experienced by groups of like-kinds involve two fungible players, each with two strategies that we tentatively^4^ label ‘C’ and ‘D’. Thus there are four possible payoffs to a focal individual (payoff matrix in Table 2).

**Table 2:** Following convention (Axelrod and Hamilton 1981), let *R* be the payoff for mutual ‘C’, *S* for unilateral ‘C’, *T* for unilateral ‘D’ and let *P* for mutual ‘D’

	C	D
C	R	S
D	T	P

Payoffs to row player are shown

All possible types of social dilemma in a two-player two-strategy symmetric game can be described by different orderings of *R*, *S*, *T* and *P* (Macy and Flache 2002):

- Prisoners’ Dilemma $$(T>R; P>S);$$
- Stag Hunt $$(R>T; P>S);$$
- Snowdrift $$(T>R; S>P);$$
- Harmony Game $$(R>T; S>P).$$

**Fig. 1:**
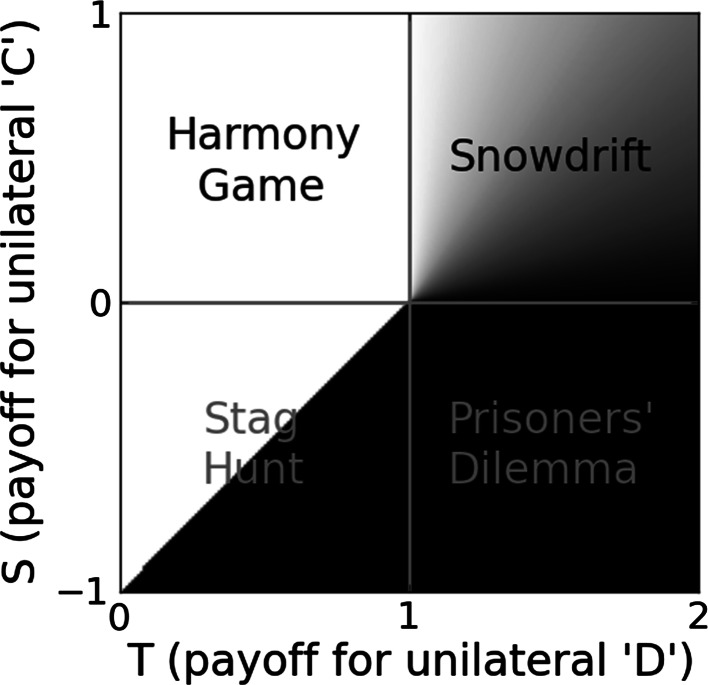
The T–S plane: conflict between individual and collective interests can be modelled as the aggregate outcome of pairwise rounds of a two-player symmetric game between individuals. The game, its dynamics and its equilibria are the determining characteristics of a social niche, as they (in conjunction with the frequencies of strategies in the population) determine the strength and direction of selection on social behaviour. The payoff matrices of these games are conventionally represented as a four-tuple of the four possible payoffs (*R*, *S*, *T*, *P*), listed in Table 2. The Stag Hunt, Prisoners’ Dilemma, Snowdrift and Harmony games are often considered separately. However, by normalising the payoff matrix so that $R=1$ and $P=0$ and limiting $(0 \le T \le 2)$ and $(-1 \le S \le 1)$, the space of all such games can be represented on a single continuous 2D plane with dimensions *S* (the payoff for unilaterally playing ‘C’) and *T* (the payoff for unilaterally playing ‘D’) (Santos et al. 2006b). *Shading* indicates equilibrium level of cooperation (*black* = 0). Synchronically, any* social niche can be characterised by a point on the T–S plane. Diachronically, social niche construction involves movement across the T–S plane. The evolution of social niches supporting cooperation requires that initially conflicted social niches be translated into instances of the Harmony Game or Snowdrift game (for full or partial cooperation respectively). Diagram adapted from Santos et al. (2006b, Fig. 2). *Any, subject to the restriction that it can be represented with a two-player two-strategy symmetric game between like-kinds

**Fig. 2:**
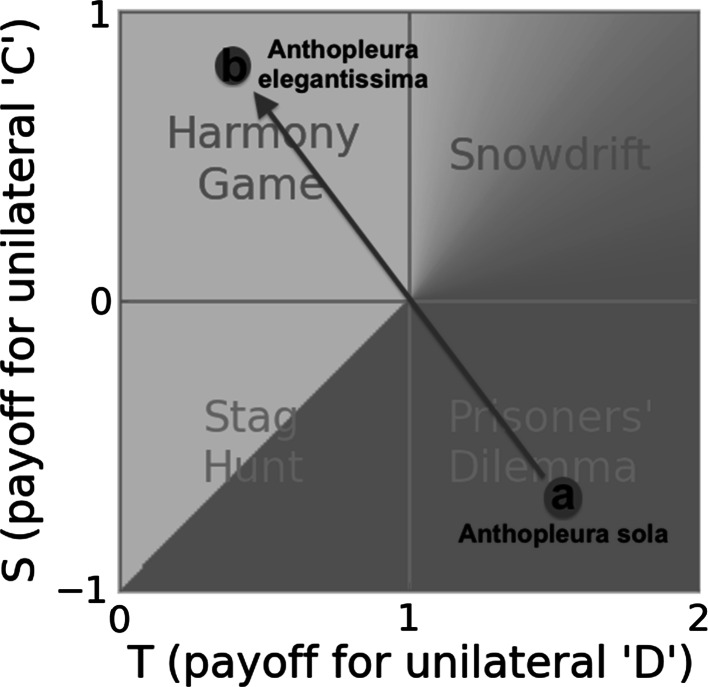
We offer a putative example of social niche construction in sea anenomes. *a* *Anthopleura sola* is a solitary-living sea anenome. Individuals compete for space on the rocky shore. It employs sexual reproduction. Offspring disperse after reproduction, so any neighbouring individuals in adjacent areas are unlikely to be close relatives. If individuals exercised growth restraint, this would allow a larger absolute biomass of *A. sola* because smaller individuals are more efficient at converting food to self. However, the cost of exercising reproductive restraint would be visited entirely upon the individual exercising it, while the benefit would be enjoyed by the whole community. They thus live in a social niche characterised by a Prisoners’ Dilemma in which the pro-social strategy is individually maladaptive and not evolutionarily stable. *b* *Anthopleura elegantissima* is a colonial anenome that shares a solitary-living common ancestor with *A. sola* (Francis 1979; McFadden et al. 1997). *A. elegantissima* colonies grow vegetatively on the benthic substrate, such that adjacent polyps are clonally related. This means individual and colony inclusive fitness interests are aligned. Colonies share club goods within the colony and antagonism in *A. elegantissima* is between colonies rather than between polyps (Ayre and Grosberg 2005). The phylogenetic tree for the Anthopleura is complex with clonality arising, being lost and arising again numerous times (Geller and Walton 2001). We tentatively suggest the changes to the social niche experienced by the polyps can be understood in terms of social niche construction, where the social niche modifying trait is one that modifies the life-history of polyps, particularly their propensity for limited dispersal after vegetative reproduction (Geller and Walton 2001)

Santos et al. (2006b) have introduced a compressed representation of the space of all such dilemmas on a single two-dimensional space^5^ that we call the ‘T–S plane’ (Fig. 1). This is a very useful tool for thinking about social evolution. The Prisoners’ Dilemma, Stag Hunt, Snowdrift and Harmony Game are often considered separately but are in fact continuous with one another. Synchronically, any^6^ social niche can be characterised by a single point on the T–S plane. Diachronically, social niche construction involves movement across the T–S plane. Figure 2 provides an example from the natural history of a colonial marine invertebrate.

### Social niche construction as movement on the T–S plane

Social niche construction was described in general terms in “Social niche construction theory employs an interactionist explanatory stance to reciprocally explain both cooperation and the factors enabling it” section. Turning back to Fig. 1, we can represent social niche construction as movement of the social niche across the T–S plane. The ways it can move depend on which SNM is acting; different SNMs act in different ways. For example, punishment reduces the gains to be made from unilateral defection, thus reducing *T* while leaving *S* unchanged. This can be represented as a shift to the left on the T–S plane. The effect of increasing assortment of strategies (perhaps by increasing genetic relatedness among interaction partners) is described fully in “Aside: the effect of assortment as SNM on various games” section. Essentially it moves any initial social niche in a straight line on the T–S plane toward the Harmony Game (point $S=1,T=0$), representing increasing toward alignment of individual and collective interests.

Consider the shading in Fig. 1. Where the initial social niche lies in a region where there is a gradient in the equilibrium level of cooperation (i.e. the Snowdrift region only), small changes in social niche immediately yield small changes in the equilibrium level of cooperation and the social niche construction process can proceed by gradual changes to the social niche. Where the initial social niche lies in a region with no gradient in the equilibrium level of cooperation (e.g. the Prisoners’ Dilemma region and the defection-dominant region of the Stag Hunt), small changes in social niche will not immediately alter the equilibrium level of cooperation. In these cases, a larger translation on the T–S plane is required. For example,^7^ consider the evolution of multi-cellularity, which has evolved independently many times (Grosberg and Strathmann 2007). In each of the land plants, the red algae, the brown algae, the animals and the fungi, the transition to multicellularity involved the evolution of a life-history involving cells ‘staying together’ after division rather than dispersing (Fisher et al. 2013). Starting among a wild type that disperses after cell division, a mutant SNM elevating the probability of adhesion after cell division would, in those cases where adhesion did occur, confer very high levels of trait assortment when compared to the freely-mixing wild type that dispersed after cell division. One need not assume this is a silver-bullet mutation that radically changes the social niche in a single generation—an evolutionary process that slightly elevated the probability of adhesion after cell division could effect such a change gradually, leading to higher and higher levels of trait assortment among the mutant strain, ultimately leading to its invasion.

### Aside: the effect of assortment as SNM on various games

There are many potential SNMs. In this subsection we concern ourselves with one that gets a lot of attention—the assortment of behaviours or traits. In the literature on the the evolution of altruism,^8^ it is widely held that positive assortment of cooperative behaviour is the key ingredient. Godfrey-Smith (2009, pp. 118–120) gives a useful review, summarising that “the familiar mechanisms behind the evolution of altruism can be seen as different ways of achieving correlation between the traits or behaviours exhibited in a population—a tendency for like to accompany like” (Hamilton 1975; Eshel and Cavalli-Sforza 1982; Michod and Sanderson 1985; Sober 1992; Skyrms 1994; Godfrey-Smith 2009; Fletcher and Doebeli 2009). Strategy assortment—the tendency for like strategies to accompany like—is indeed a powerful SNM, biologically instantiated in a number of ways, including relatedness. But while positive assortment of cooperative traits is indeed sufficient for pro-social behaviour to be stable in a Prisoners’ Dilemma, this is not true of all two-player two-strategy symmetric games.

For games where $S<P$ (the Stag Hunt and Prisoners’ Dilemma), there is no direct benefit to the actor from its own unilateral playing of ‘C’, so ‘C’ can only be selected for if it is reliably reciprocated. In the Prisoners’ Dilemma this means that for ‘C’ to be selected for, there must be circumstances that cause there to be positive assortment of cooperation. (Recall that we’re restricting our discussion to assortment as a SNM in this subsection. Other SNMs such as side-payments or punishment are not considered here.) In the Stag Hunt, if the balance between the payoffs and the frequency of cooperators dictates that ‘D’ is favoured by selection, then for ‘C’ to evolve from those conditions there must be positive assortment of cooperation (Skyrms 2004, Postscript). These are the cases where positive assortment of cooperative behaviour is necessary (and where ‘C’ might reasonably be called altruism). In Stag Hunts where the standing frequency of cooperators in the population is sufficiently high, it may be worth chancing it even without any mechanisms causing positive assortment.

There is another class of games: those where the benefit accruing to an actor playing ‘C’ exceeds the cost of creating it (i.e games where $S>P$). This class includes all Snowdrift (Hawk–Dove) games. Pepper’s (2000) example of games involving whole-group beneficial traits that provide more benefit to the actor than they cost (even though the benefit is shared with others) are in this category. In such social niches, positive assortment of cooperation is not necessary to make the ‘C’ trait evolutionarily stable at some non-zero level. ‘C’ will be viable at some non-zero equilibrium level (called the ‘mixed-strategy equilibrium’) in virtue of it providing direct benefit to the actor, even in the absence of positive assortment. (In these cases, ‘C’ might reasonably be called mutual cooperation.) While positive assortment is not necessary in these cases, it is sufficient if it does obtain: any external factor increasing positive assortment in a Snowdrift game (where $S+T<2R$) will increase the level of cooperation and collective welfare. In the case of Snowdrift games where $S+T>2R,$ it is not the case that collective welfare increases with positive assortment of strategies, or with greater frequency of the ‘C’ strategy. In these interesting cases, division of labour between the two strategies produces the best collective payoff and increasing positive assortment can be detrimental (Tudge and Watson 2015). It is inappropriate to call the ‘C’ strategy cooperation in such cases.

**Fig. 3:**
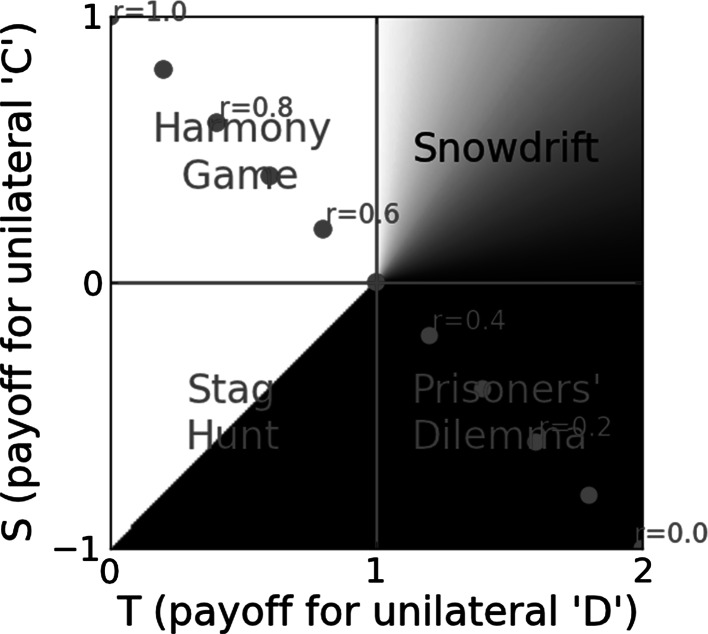
Relatedness as a social niche modifier: social niche modifiers alter the direction and strength of selection on social behaviour. Between like-kinds, genetic relatedness is usually the most important social niche modifier. (Between unlike-kinds it is typically something else, such as a life-history involving co-dispersal of mutualists.) The figure shows the effect of relatedness, *r*, as a social niche modifier among conspecifics. When games are played between relatives, the expected payoffs are modified due to the elevated possibility that the interaction partner may play the same strategy as the focal individual (Grafen 1979). In a social niche that between non-kin ($r=0$) would be a Prisoners’ Dilemma, the effective game being played between diploid full sibs $\left(r=\frac{1}{2}\right)$ is less conflicted and a game played between clones ($r=1$) is not conflicted at all

Modifying a social niche by modifying assortment has a quantifiable effect on the effective game being played, with a geometric interpretation on the T–S plane (see Fig. 3). To see this, consider the following reasoning due to Jackson and Watson (2015). Let degree of assortment $\alpha$ mean that with probability $\alpha$ an individual will play another with the same ST as itself while with probability $(1-\alpha )$ it will play a randomly selected (non-self) member of the population. When $\alpha =1,$ the game being played is the Harmony Game $$ \left( {\begin{array}{cc} R &{}\quad R \\ P &{}\quad P \end{array}} \right) $$, when $\alpha =0$, the game being played is the unmodified underlying game $$ \left( {\begin{array}{cc} R &{}\quad S \\ T &{}\quad P \end{array}} \right) $$ and when $\alpha$ is between 0 and 1 the effective game is $$ \left( {\begin{array}{cc}R &{}\quad S+\alpha (R-S) \\ T+\alpha (P-T) &{}\quad P \end{array}} \right) $$. This has the neat geometric analogue on the T–S plane of assortment interpolating along the straight line between the point (*T*, *S*) when there is no assortment to the point (0, 1) when there is full assortment. So an underlying game plus assortment is formally equivalent to another game in a freely-mixed population. A similar result for relatedness (an instantiator of ST assortment) is given by Taylor and Nowak (2007).

## Social niche construction can explain evolutionary transitions in individuality

It has long been recognised that the biological world has a part-whole hierarchical structure and that entities at several levels in that hierarchy could, in principle, be evolutionary units in virtue of potentially having heritable variation in fitness (Lewontin 1970). The debate about the relative strength of the evolutionary process at each of these levels (e.g. Williams 1966; Wilson 1975b; Dawkins 1976; Vrba 1984) does not address the origin of the hierarchy itself (Griesemer 2000). Explaining the evolution of new hierarchical levels at which the evolutionary process acts is the business of the Major Transitions research programme (Buss 1987; Maynard Smith 1988; Maynard Smith and Szathmáry 1995; Michod 1999). Here, the thing to be explained is not whether this or that level is the ‘real’ or ‘main’ level of selection but how the levels arose in the first place. Okasha (2005) describes the earlier levels-of-selection question as formulated in a ‘synchronic’ way, while the major transitions approach is to formulate the question in a ‘diachronic’ way. It is to this diachronic question that social niche construction theory offers an answer.

In more detail, the thing to be explained is as follows. Prior to a transition,^9^ particles are the units of evolution,^10^ in the sense that they have the properties of heritable variation in fitness (and collectives or groups of them do not). After a transition, collectives (consisting of particles) are the units of evolution, in the sense that they have the properties of heritable variation in fitness (and, in this idealised version, particles do not). There is another way to say this, using Price’s covariance formalism (Price 1970; Okasha 2006). Prior to a transition we find a non-zero covariance between particles’ fitnesses and the character of their offspring. After a transition, particles’ fitnesses do not covary with the character of their offspring but we do find a non-zero covariance between collectives’ fitnesses and the character of their offspring collectives. The challenge is to explain how an evolutionary process could bring about such a transition.

We began this paper by characterising social evolution theory as primarily employing an externalist explanatory stance. Recall that externalism has it that the properties of biological entities (including social behaviours such as cooperation) are to be explained in terms of their adaptation by natural selection to factors external to them. We now assess how well the externalist stance handles the challenge to explain how collectives become units of evolution (and how particles lose this property) during evolutionary transitions.

There is no doubt that many collective life-forms have striking features that suppress internal conflict (Table 1 lists many examples). Some have tried to meet the challenge by invoking an externalist explanation that casts collectives in the role of units of evolution (i.e. ‘collective-level selection’, properly understood). Roughly, the story is that collectives have properties that suppress within-collective conflict. Collectives are differentially fit according to the extent to which they are successful in within-collective conflict suppression. Less conflicted collectives are fitter and so the conflict suppression mechanisms evolve as adaptations of collectives. This sort of reasoning can be found in works of natural history that try to explain the existence of conflict-suppression mechanisms (Wilson and Sober 1989; Wilson and Hölldobler 2005; Wilson and Wilson 2007) and in modelling work that pre-supposes a collective-level evolutionary process and then finds that it acts to strengthen conflict-suppression (Michod and Roze 1999; Michod 2006). However, we find it problematic because it invokes a product of a collective-level evolutionary process to explain how the collective level became a level at which the evolutionary process operates. As such, it is not consistent with our understanding of the relation between causes and effects. This sort of reasoning contravenes Williams’ Principle (Williams 1992; Sober and Wilson 1998; Okasha 2006, p. 113), which states that collective-level adaptations are evidence of past collective-level selection and not a prior condition of it. As Okasha (2006, p. 225) says, “although some collective-level phenomena might usefully be explained ‘at their own level’, the original evolution of the collectives is not one of them.”

Perhaps we can instead explain what makes collectives into evolutionary units by taking an externalist explanatory stance at particle level instead? Recall that such a stance explains social behaviours (such as cooperation) as an evolutionary response to some external factor (what we call a social niche). It is hard to see how such a project could proceed, given that the suppression of the evolutionary process at particle-level (where there would be selection for ‘selfishness’) has been taken to be central to the whole task of explaining major transitions since the outset. As Maynard Smith (1988) asked, “How did natural selection bring about the transition from one stage to another, since, at each transition, selection for ‘selfishness’ between entities at the lower level would tent to counteract the change?”.

Social niche construction theory does not suffer from either of the two problems above. It does not invoke a collective-level evolutionary process to explain how collectives became units of evolution. Nor does it assume that particle-level selection must always be opposed to the fitness interests of collectives, because it allows that particles can partly construct their own social niche. Social niche construction explains the advent of a collective-level evolutionary process as a side-effect of a particle-level evolutionary process that aligns the fitness interests of particles within collectives as a means to making them fitter.

To see how, consider Price’s covariance formalism again. Evolutionary change occurs in a population of entities when there is a non-zero covariance between those entities’ fitnesses and the character of their offspring. We can break this down into a pair of conditions,^11^ one about selection and one about heredity. Evolutionary change occurs in a population of entities when there is:

1. covariance between entity fitness and entity character (selection);
2. covariance between entity character and mean offspring character (heritability).

Before an evolutionary transition, particles meet conditions 1 and 2 by definition. Before an evolutionary transition, collectives fail condition 1 because there is no covariance between their fitnesses and their characters. Before an evolutionary transition, collectives fail condition 2 because within-collective selection denies collective-level heritability. This is because within-collective selection changes the distribution of particle types over the lifetime of a collective, such that the particles it consists of at maturity (and that will go into its propagules, howsoever formed) will not have the same type distribution as those from which it was itself founded. After an evolutionary transition, collectives meet conditions 1 and 2 by definition. Particles fail condition 1 (they do not vary in fitness within their collectives, so there can be no co-variance) but still meet condition 2 (there is still particle-level heritability). As we saw in “Social niche construction theory employs an interactionist explanatory stance to reciprocally explain both cooperation and the factors enabling it” section, social niche construction can be understood in terms of the evolution of cooperation among particles, so aligning within-collective fitness interests. But if we think of that same process in terms of (1) selection and (2) heritability at both levels, we can now see that, for particles, the alignment of fitness interests within collectives is also the reduction of fitness variance within collectives (and hence any character-fitness covariance) and thus the negation of condition 1 for particles. All particles in a collective get the same fitness. Simultaneously, for collectives, that same process involves an increase in character-fitness covariance among collectives. The character here is the conflict suppression mechanisms—the SNM and fitness is a function of the extent to which the constituent particles cooperate. Finally, that same social niche construction process also increases the extent to which collective-level heredity obtains (condition 2), by suppressing within-collective change. So while there is no selection-for collectives being the unit of evolution, there is selection-of the conditions for them to be so [selection-of/selection-for as per Sober (1984)].

Thus we offer social niche construction as an explanation for how collective-level selection could get started and thereby how evolutionary transitions in individuality might be enabled. Of course, once there is a collective-level selection regime in force then a collective-level evolutionary process can evolve division of labour, specialisation of parts and all manner of complex adaptations.

## Footnotes

1 The externalist explanatory stance is captured succinctly by Williams (1992, p. 484) when he says “Adaptation is always asymmetrical; organisms adapt to their environment, never vice versa.”

2 In what follows, we will call these factors ‘social niche modifiers’.

3 See Archetti (2009) and Archetti and Scheuring (2012) for discussion of some more biologically realistic alternatives to two-player symmetric games—all of which are more rather than less amenable to cooperation.

4 Whether playing ‘C’ can be properly described as ‘cooperation’, ‘strong altruism’ or something else depends on further details of the game being played—see “Aside: the effect of assortment as SNM on various games” section.

5 See also Weibull (1995, p. 29).

6 Subject to our stated assumption that the social niche can be modelled by aggregating a two-player two-strategy symmetric game.

7 We thank an anonymous reviewer for prompting us to include this example.

8 ‘The evolution of altruism’ and ‘the evolution of cooperation’ are sometimes distinguished in a principled way (not always consistently) and sometimes run together. Which term is correct depends on the underlying game implicit in each case. We give a full account below.

9 We treat ‘major evolutionary transition’ and ‘evolutionary transition in individuality’ as synonyms.

10 There is a risk of confusion over the term ‘level of selection’. It is often assumed to mean ‘level at which there is heritable variation in fitness’ (Lewontin 1970; Keller 1999; Okasha 2006) but taken literally it does not say anything about heritability. (For example, in MLS1 trait-group models, particles and groups are both units of selection in the strict sense but only particles have heritability while groups do not.) Following Maynard Smith (1987, p. 121), we prefer the term ‘unit of evolution’ over ‘unit of selection’, because the former implies a unit with heritable variation in fitness (which is what we want it to mean) while the latter is dangerously ambiguous.

11 As Okasha (2006, p. 37) points out, the equivalence between the single-part version of the Price formalism and the two-part version is not fully general because covariance is not generally a transitive relation. Our two-part price formalism should be understood as assuming that there is covariance between entity fitness and offspring character.
